# The Use of DNA Barcoding on Recently Diverged Species in the Genus *Gentiana* (Gentianaceae) in China

**DOI:** 10.1371/journal.pone.0153008

**Published:** 2016-04-06

**Authors:** Juan Liu, Hai-Fei Yan, Xue-Jun Ge

**Affiliations:** 1 Collaborative Innovation Center of Jiangxi Typical Trees Cultivation and Utilization, Jiangxi Agriculture University, Nanchang, China; 2 Key Laboratory of Plant Resources Conservation and Sustainable Utilization, South China Botanical Garden, the Chinese Academy of Sciences, Guangzhou, China; Chinese Academy of Medical Sciences, Peking Union Medical College, CHINA

## Abstract

DNA barcoding of plants poses particular challenges, especially in differentiating, recently diverged taxa. The genus *Gentiana* (Gentianaceae) is a species-rich plant group which rapidly radiated in the Himalaya-Hengduan Mountains in China. In this study, we tested the core plant barcode (*rbcL* + *matK*) and three promising complementary barcodes (*trnH-psbA*, ITS and ITS2) in 30 *Gentiana* species across 6 sections using three methods (the genetic distance-based method, Best Close Match and tree-based method). *rbcL* had the highest PCR efficiency and sequencing success (100%), while the lowest sequence recoverability was from ITS (68.35%). The presence of indels and inversions in *trnH-psbA* in *Gentiana* led to difficulties in sequence alignment. When using a single region for analysis, ITS exhibited the highest discriminatory power (60%-74.42%). Of the combinations, *matK* + ITS provided the highest discrimination success (71.43%-88.24%) and is recommended as the DNA barcode for the genus *Gentiana*. DNA barcoding proved effective in assigning most species to sections, though it performed poorly in some closely related species in sect. *Cruciata* because of hybridization events. Our analysis suggests that the status of *G*. *pseudosquarrosa* needs to be studied further. The utility of DNA barcoding was also verified in authenticating ‘Qin-Jiao’ *Gentiana* medicinal plants (*G*. *macrophylla*, *G*. *crassicauli*s, *G*. *straminea*, and *G*. *dahurica*), which can help ensure safe and correct usage of these well-known Chinese traditional medicinal herbs.

## Introduction

DNA barcoding, a term first proposed by Hebert in 2003 [1], has developed as a rapid and reliable technology to identify species based on variation in the sequence of short standard DNA region(s). This tool is now successfully used in a variety of biological applications, including discovering cryptic species [2], detecting invasive species [3], reconstructing food webs [4] and identifying medicinal plants in mixtures [5, 6]. In 2009, the Plant Working Group of the Consortium for the Barcode of Life (CBOL) proposed the combination of *rbcL* and *matK* as a ‘core barcode’ for identification across land plants [7]. However, the application of DNA barcoding has been hindered by the difficulty of distinguishing closely related species, especially in recently diverged taxa [8]. Limited performance of *rbcL* + *matK* has been reported in many complex taxa [9–13]. Since the plastid intergenic spacer *trnH-psbA* and the nuclear ribosomal internal transcribed spacer ITS/ITS2 have been proposed as supplementary barcodes for land plants [14–16], the evaluation of plant barcoding regions has focused on the performance of these five loci (*rbcL*, *matK*, *trnH*-*psbA*, ITS and ITS2) individually and in various combinations. For example, *matK* + ITS was recommended as the barcode to be used in the genus *Primula* [17], while ITS + *trnH-psbA* + *matK* was demonstrated as the best barcode for discriminating *Rhododendron* species [18]. More studies are still needed to assess the efficacy of plant barcodes in closely related species, especially for groups that diverged recently.

Floristic DNA barcoding has been demonstrated to be effective for identifying species in species-rich regions that would otherwise require detailed ecological study for characterization [19]. However, correctly identifying the species of complex genera in local flora can still pose a significant challenge in biodiversity hotspots. For example, poor species resolution was found for sister species in the genera *Crocus* and *Quercus* in African forests [20], and low sequence variation was demonstrated for most polytypic genera in the Dinghushan subtropical forests of China [21]. This is largely due to the frequent occurrence of close relatives in the distribution centers of large genera.

*Gentiana* L. (Gentianaceae) consists of 361 species with a subcosmopolitan distribution; more than half of all the species are found in southwestern China and the adjacent northeastern Himalaya-Hengduan Mountain region [22]. This region is considered the center of diversification of many plant genera, such as *Rhododendron*, *Primula*, *Pedicularis* and *Gentiana* [18]. The genus *Gentiana* is divided into 15 sections, of which 5 are further divided into 22 series [23]. Although the monophyly of several sections has been verified, the taxonomic treatment of species in each section is still controversial, since the radiation occurred only recently and so has resulted in little variation in morphological features [23, 24]. Molecular studies suggest that rapid evolutionary processes have occurred in at least two sections: *Chondrophyllae* and *Cruciata* [24–26].

Most of the species in these sections are distributed in the mountainous regions of southwestern China and the nearby Qinghai-Tibet Plateau. Sect. *Chondrophyllae* is the largest and the most widely distributed section in this genus; it consists of about 163 species and is divided into 10 series [23]. The identification of individual species in this section is by no means an easy task due to the high morphological variability of the small annual or biennial plants [25]. Sect. *Cruciata* contains 21 perennial species. Some species in this section may have diverged four million years ago, though most result from more recent speciation events [26]. Twelve species in sect. *Cruciata* are well known in traditional Chinese medicine and are also widely utilized for medicinal purposes in Asia (e.g. *Gentiana davidii* as drugs of cholesteric and hepatic diseases) [27, 28]. Their dried roots are used as medicinal materials, and adulterants are frequently detected in traditional medicinal markets [29]. Authenticating medicinal plants can be very difficult because of similarities in morphological appearance [28–31]. Finding an appropriate DNA barcode to discriminate *Gentiana* species would therefore be invaluable.

In this study, five DNA barcoding candidate regions (*rbcL*, *matK*, *trnH-psbA*, ITS and ITS2) were chosen for evaluation. We aimed to: i) evaluate the discriminative ability of the five barcoding regions, *rbcL*, *matK*, *trnH-psbA*, ITS and ITS2 and their combinations; and ii) explore the efficacy of DNA barcoding in *Gentiana*.

## Materials and Methods

### Taxon Sampling

We collected 79 accessions comprising 1–8 individuals of 30 species from China. In addition to sect. *Chondrophyllae* (12 species) and sect. *Cruciata* (8 species), the following 4 sections were also selected for analysis: sect. *Frigidae* (2 species), sect. *Microsperma* (2 species), sect. *Kudoa* (4 species) and sect. *Monopodiae* (2 species). All specimens were collected from the wild and no specific permissions were required for the corresponding locations/activities. The field studies did not involve endangered or protected species. Vouchers specimens of the collected taxa were deposited in the South China Botanical Garden Herbarium (IBSC) (S1 Table).

### PCR and Sequencing

Genomic DNA was extracted from dried leaves in silica gel using the CTAB method [32]. Four regions (*rbcL*, *matK*, *trnH-psbA* and ITS), were amplified and sequenced to test the effectiveness of their primers in *Gentiana*. *matK* required the use of two primer pairs (*matK*-3F-Kim/-xf [9] and *matK*-xf/-5r [33]). The other three regions each used one universal pair of primers for sequence amplification (*rbcL-*Rev/-For, trnH05/psbA3 and ITS4/ITS5). A 25 μl PCR reaction mixture was prepared and amplified according to the procedure described by Zhang *et al*. [9]. PCR products were purified using a DNA gel cleaning kit (Takara) and sequenced in both directions on an ABI3730X sequencer (Applied Biosystems, USA) using the amplification primers. All sequences were deposited in GenBank (S1 Table).

### Data analysis

The original trace files were checked and verified by searches with NCBI’s web-based BLASTn. Sequences were assembled and inspected with Sequencher 4.1 [34], aligned with the MUSCLE aligner implemented in Mega 5.0 [35], and modified manually using Se-al version 2.0a11 [36]. Due to the presence of indels, *trnH-psbA* was aligned by section, and intraspecific inversions were found in this region. To reduce costs, we retrieved ITS2 from ITS data and re-amplified failed ITS samples.

Genetic divergence was calculated for the five markers according to the Kimura 2-Parameter (K2P) model using MEGA 5.0. Six distance parameters were estimated, including three inter-specific distance parameters (average inter-specific distance, average theta prime and smallest inter-specific distance) and three intra-specific parameters (average intra-specific distance, theta and largest intra-specific distance) [15]. We calculated the mean K2P distances for each of the six sections and explored the difference in evolutionary divergence among sections in *Gentiana*.

Three methods, namely a genetic distance-based method, the analysis of Best Close Match and a tree-based method, were employed to evaluate the five single markers and their combinations. The first two methods were conducted using the R package SPIDER [37]. For the tree-based method, two phylogenetic trees were inferred to calculate the rate of monophyletic clusters. Neighbor-Joining (NJ) trees were built using the software PAUP* version 4b10 with the K2P model. Node supports were assessed by 1000 bootstrap replicates. A Bayesian inference (BI) analysis was implemented using MrBayes on XSEDE (v3.2.6) [38], and the optimal models for each marker were determined under the Akaike Information Criterion (AIC) using jModelTest2 on XSEDE (v2.1.6) [39]. Both were conducted on the CIPRES supercomputer cluster [40] with parameter sets according to Yan *et al*. [41]. Species were considered successfully identified if the monophyletic cluster of sequences representing a species was grouped with a bootstrap value above 70% or a posterior probability above 0.95. Singleton species (species with one specimen) were included and considered as the source of resolution failure.

### Sequence acquisition from GenBank

In order to minimize the bias from incomplete sampling, we expanded our dataset using data from public databases. Since limited DNA barcode data is available for the genus *Gentiana*, we retrieved all Gentianaceae sequences involving *rbcL* or *matK* and all *Gentiana* sequences with the internal transcribed spacer (ITS and ITS2) from GenBank. Sequences available on NCBI may not necessarily link with taxonomically validated voucher specimens, so we examined all the sequences downloaded from Genbank in an effort to ensure correct species identification. We found that almost all of the *Gentiana* sequences available in Genbank are associated with published phylogenetic or barcoding papers, and the sources of the sequences were identified by specialists working on this genus. Collection information for the voucher specimens was present in the relevant papers.

Due to difficulties in sequence alignment, *trnH-psbA* was not used for further analysis. We also removed sequences less than 300 bp in size and those lacking clear *Gentiana* species identification. We followed an established pipeline [42] to remove fungal sequence contamination. In some cases multiple individuals were available from a single population but we analyzed only two sequences due to time constraints. The whole dataset comprised of 280 sequences for *rbcL*, 274 sequences for *matK*, 243 sequences for ITS, and 304 sequences for ITS2. The data were analyzed with tree-based analysis, as above.

## Results

### Sequence recoverability and divergence

*rbcL* had the highest PCR efficiency and sequencing success (100%), followed by *matK* (96.2%) and *trnH-psbA* (96.2%) (Table 1). Sequence recoverability was lowest for ITS (68.35%) because of the incongruence of multiple copies which resulted in some ‘messy’ sequences. ITS2 had 16 more sequences, and its sequence recoverability was 88.61%. Due to the presence of indels, the length of *trnH-psbA* varied from 199 to 486 bp in different species, leading difficulties in sequence alignment (total length of 698 bp). In total, 355 sequences were obtained and submitted to the GenBank database (S1 Table).

**Table 1:** Sequence recoverability of the five barcodes.

	Recovered Species	Recovered Samples	Recoverability	Sequencing length	Aligned length	Variable characters
*rbcL*	30	79	100%	534–564	554	55
*matK*	30	76	96.2%	809–855	743	220
ITS	26	54	68.35%	634–712	636	190
ITS2	29	70	88.61%	197–229	227	89
*trnH-psbA*	30	76	96.2%	199–486	698	240

Comparative analysis of inter- versus intra-specific distances for the five regions was conducted using six parameters [15] (Table 2). *trnH-psbA* exhibited the highest interspecific genetic distance, followed by ITS2, *matK*, ITS and *rbcL*. For the divergence among conspecifics, the rank order of theta was *trnH-psbA*, ITS2, ITS, *matK* and *rbcL*. An ideal barcode should possess higher interspecific variation than intraspecific variation in order to distinguish different species. ITS had one of the smallest interspecific distances and a relatively low coalescence depth.

**Table 2:** Six genetic distance parameters measured with the five barcodes.

	Interspecific distance			Intraspecific distance		
	Mean (SD)	Theta Prime (SD)	Smallest (SD)	Mean (SD)	Theta (SD)	Coalescent Depth (SD)
*rbcL*	0.05602 (0.02531)	0.05715 (0.01045)	0.00963 (0.01466)	0.00264 (0.00563)	0.00191 (0.00389)	0.00357 (0.00357)
*matK*	0.06901 (0.03357)	0.06707 (0.01235)	0.01141 (0.01489)	0.00337 (0.0060)	0.00172 (0.0034)	0.00373 (0.00722)
ITS	0.05829 (0.02650)	0.05981 (0.01695)	0.01886 (0.02367)	0.00332 (0.00698)	0.00461 (0.11374)	0.00585 (0.01148)
ITS2	0.07429 (0.0320)	0.07644 (0.02089)	0.01873 (0.02465)	0.00814 (0.01629)	0.00540 (0.01286)	0.00976 (0.02153)
*trnH-psbA*	0.10987 (0.05400)	0.11218 (0.01700)	0.01411 (0.01502)	0.01021 (0.02124)	0.00583 (0.01141)	0.01090 (0.02201)

### Species discrimination

A genetic distance-based method, the Best Close Match and a tree-based method were used to evaluate the discriminatory power of barcodes in *Gentiana*. In the single region analysis, *rbcL* performed poorly, as expected (Table 3). The highest discriminatory power was obtained using ITS (60.0%-74.42%), followed by *trnH-psbA* (45%-71.21%), *matK* (52.63%-69.23%) and ITS2 (50%-67.80%). When combining two barcodes, *matK* + ITS gave the highest discrimination success (71.43%-88.24%). The three-region combination of *rbcL* + *matK* + ITS achieved slightly higher species identification success than *matK + ITS* when using the Best Close Match method (87.18%).

**Table 3:** Species resolution using a genetic distance-based method, the Best Close Match method and the tree-based method with five barcodes and their combinations.

	Genetic distance (%)	Best Close Match				NJ trees (%)	BI analysis (%)
		Correct (%)	Ambiguous (%)	Incorrect (%)	No ID (%)		
*rbcL*	45.57	59.42	37.68	2.90	0	50.0	40.0
*matK*	65.79	69.23	18.46	6.15	6.15	57.89	52.63
ITS	70.37	74.42	18.60	2.32	4.65	73.33	60.0
ITS2	58.57	67.80	22.03	3.39	6.78	50.0	50.0
*trnH-psbA*	50	71.21	19.70	3.03	6.06	45.0	60.0
*rbcL+matK*	63.17	72.31	18.46	6.15	3.08	63.16	52.63
*rbcL+*ITS	82.35	84.16	7.69	2.56	5.12	66.67	66.67
*rbcL+*ITS2	77.61	83.64	9.09	3.64	3.64	55.56	55.56
*rbcL+trnH-psbA*	58.90	74.19	22.48	1.61	1.61	60.0	50.0
*matK+*ITS	88.24	84.61	0	7.69	7.69	71.43	71.43
*matK+*ITS2	79.10	80.0	0	12.73	7.27	70.58	64.71
*matK+trnH-psbA*	63.10	69.35	14.52	9.68	6.45	47.36	68.42
*rbcL+matK*+ITS	88.24	87.18	0	7.69	5.13	71.43	71.43
*rbcL+matK*+ITS2	82.89	89.09	0	9.09	1.82	64.71	64.71
*rbcL+matK+trnH-psbA*	63.01	72.58	14.52	9.68	3.23	60.0	60.0

Analysis of the GenBank data showed that *rbcL* and *matK* can very reliably assign sequences to the genus *Gentiana* (100% success rate). Moreover, the DNA barcoding markers performed well at the section level. *rbcL* grouped 5/9 sections correctly, *matK* identified 8/10 sections, ITS identified 7/11 sections and ITS2 identified 5/11 sections (S1 Fig).

### Comparative analysis of DNA barcoding identification among different sections

**Fig 1:**
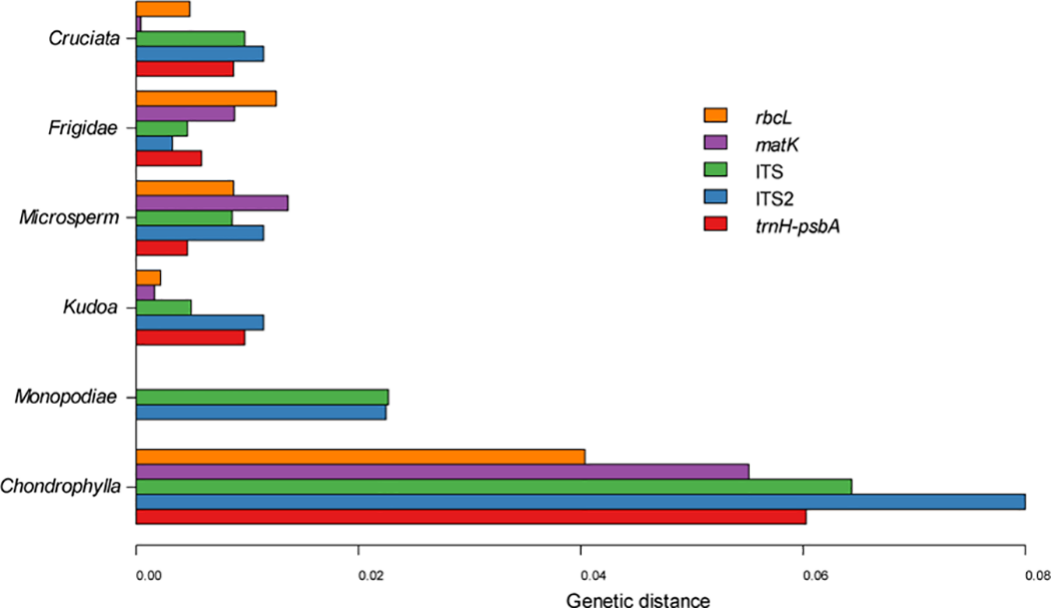
Genetic distance within sections for the five barcodes.

**Fig 2:**
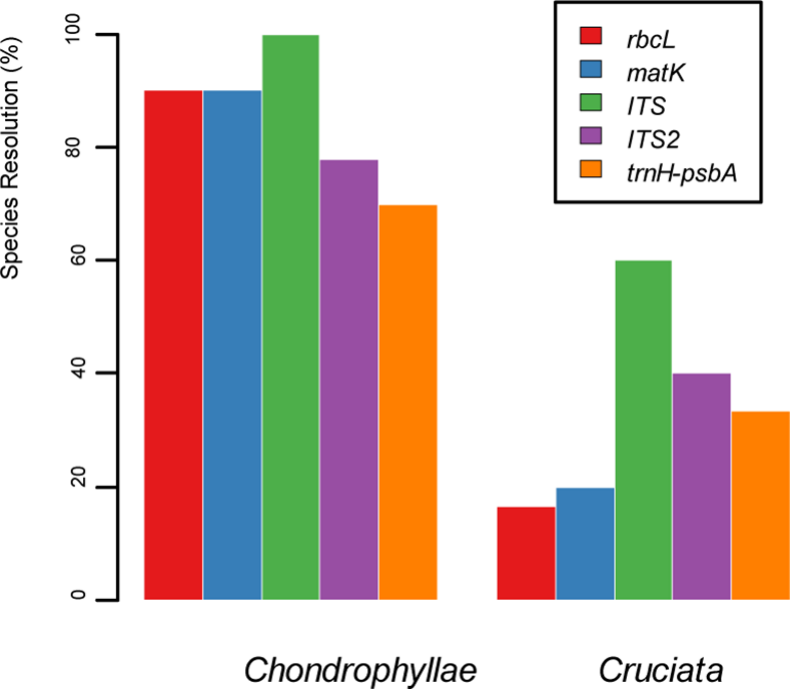
Species resolution comparison using NJ-tree analysis between sect. *Chondrophyllae* and sect. *Cruciata*.

When the comparative analysis was restricted to each section, the mean K2P distances of all the barcodes showed significant heterogeneity (Fig 1). Divergences in sect. *Chondrophyllae* were significantly higher than in the other five sections, particularly sect. *Cruciata*, where the divergences were four times lower. There were significant ‘barcoding gaps’ for all barcodes in sect. *Chondrophyllae*, but no gap existed in sect. *Cruciata* (S2 and S3 Figs). The species identification rate in sect. *Chondrophyllae* was 72.72%, regardless of whether chloroplast or nuclear regions were used (Fig 2). In sect. *Cruciata*, the nuclear regions (ITS, ITS2) performed much better (62.50%-12.50%) than chloroplast markers (37.50%-12.50%).

## Discussion

### The performance of DNA barcodes in *Gentiana*

While *rbcL* and *matK* have been proposed as core DNA barcodes for plants [43], their disadvantages (the low genetic divergence of *rbcL* and poor primer universality of *matK*) have since been reported in many studies [9, 11, 13, 44, 45]. In the present study, we did not encounter primer problems with *matK*, since we employed two universal pairs of primers recommended by CBOL. However, the combination of *rbcL* and *matK* had the lowest genetic divergence and could only poorly discriminate species in this genus. In sect. *Cruciata*, *rbcL + matK* was even less effective for differentiating closely related species (0–32.5%). The poor performance of *rbcL + matK* has been reported in many species-rich plant groups, such as *Lysimachia* (47.1%-60.82% discriminatory power) [9], *Berberis* (15.4%-23.1%) [46], *Viburnum* (53%) [12], *Primula* L. sect. *Proliferae* (50%) [13]. Therefore, as in other species-rich genera, the core barcodes *rbcL + matK* must be supplemented with more effective barcodes for the genus *Gentiana*.

The high variation of the *trnH-psbA* spacer and the availability of a universal primer have led to its successful application in many DNA barcoding studies. In this study, *trnH-psbA* gave the highest inter- and intra-specific divergence of all the single regions. Nevertheless, several problems limit its use in *Gentiana*. First, extensive variation in the size of *trnH-psbA* resulted in alignment ambiguities. The length of the *trnH-psbA* sequence varied across five of the six sections; the sequence was 199–327 bp in sect. *Chondrophyllae*, 410–486 bp in sect. *Cruciata*, 413 bp in sect. *Monopodiae*, 348–442 bp in sect. *Microsperm*, 397–470 bp in sect. *Kudoa*, and 360–391 bp in sect. *Frigidae*. We therefore had to manually adjust the algorithmically-generated alignment, which required significant effort. Second, a short 30 bp inversion was detected in *trnH-psbA* in *Gentiana*. Frequent inversions of *trnH-psbA* in the family Gentianaceae were reported by Whitlock *et al*. [47]. Furthermore, we found a 21 bp inverted repeat region which may form a stem loop and facilitate the process of inversion [48]. Re-inversions were found in *G*. *panthaica* (9 bp) and *G*. *pseudosquarrosa* (12 bp). These inversions lead to overestimation of the variation between closely related species and unite distantly related taxa, resulting in erroneous phylogenetic inferences [47]. Third, mononucleotide repeats (poly A/T) in bi-directional reads from *trnH-psbA* have proved a hindrance to obtaining full length sequences in many other studies [9, 43], although this was not the case in our study. An ideal universal DNA barcode should be standardized and easily accepted by non-experts [49]. While *trnH-psbA* may serve as a valuable DNA barcode in plant groups where these challenges are not encountered, too much effort must be spent resolving these problems in *Gentiana*; *trnH-psbA* is therefore not recommended as a DNA barcode for *Gentiana*.

ITS has been proposed as a DNA barcode because it evolves 3–4 times faster than the plastid regions, and it has been successfully used in many phylogenetic studies [49]. A previous study of *Gentiana* at the sectional level demonstrated the phylogenetic utility of ITS sequences [22]. Many taxonomy-based barcoding studies have demonstrated its effectiveness as a barcode for identifying species, even in complex taxa, such as *Ficus* [10], *Lysimachia* [9] and *Viburnum* [12]. In this study, ITS exhibited the best performance of all five barcodes for discriminating species of *Gentiana*. This region was also capable of differentiating the closely related species *Gentiana dahurica*, *G*. *decumbens* and *G*. *macrophylla*. However, the sequencing success of ITS in *Gentiana* was low (63.7%), which may imply incomplete concerted evolution of a nuclear multiple-copy locus in this genus.

A short nuclear region (300 bp) of ITS2 was proposed as a novel DNA barcoding region for medicinal plants by Chen *et al*. [15] and has been strongly recommended for many groups [42, 50–52]. ITS2 is favored as a barcode because it can be amplified with a universal primer and is easy to sequence. Compared with the full length ITS, ITS2 may be less powerful for differentiating closely related species [53]. However, in our study, ITS2 sequences were obtained from more 16 individuals than ITS sequences. Moreover, the short length of this region makes it potentially more attractive for actual DNA barcoding applications, such as barcoding degraded DNA from the powder of herbal products [6] and ‘DNA meta-barcoding’ using Next Generation Sequencing [54].

No single barcode was perfect for species identification in *Gentiana*. The use of multiple regions with more and less variation, such as combinations of plastid regions and nuclear regions, has been recommended and has been shown to improve discriminatory power in many barcoding studies [12, 16]. In the present study, the combination of the two regions *matK* + ITS gave the highest species resolution (88.24%), roughly equivalent to the resolution of the three-marker combination *rbcL* + *matK* + ITS. We support the recommendation of the *matK* + ITS combination as a core DNA barcode for large genera of flowering plants [16]. *matK* + ITS seems to be the best choice as a DNA barcode for *Gentiana*, with ITS2 serving as a back-up region for ITS to improve sequence recoverability.

However, other highly variable regions are required to successfully identify all species. In a recent *Gentiana* barcoding study, 5S rRNA and *trnL-F* were successfully used as barcodes to differentiate five medicinal *Gentiana* species and their adulterants [55]. However, using 5S rRNA requires cloning the amplified PCR product rather than direct sequencing, which limits its value as a barcode. *trnL-F*, with universal primers and high discriminatory power, also seems to be a good choice as a barcode for *Gentiana* species and has been suggested for other groups that have undergone a recent radiation [50], but more samples should be tested to validate this conclusion in *Gentiana*.

### Species resolution comparison between sect. *Chondrophyllae* and sect. *Cruciata*

Although sect. *Chondrophyllae* and sect. *Cruciata* have both recently diverged in the Himalaya-Hengduan Mountains [22, 25], greater discrimination success was achieved for sect. *Chondrophyllae* (72.72%-83.33%) than for sect. *Cruciata* (0–62.5%) (Fig 2). This may be partly attributed to life history traits which are correlated with the molecular evolution rate [56]. Tall perennial plants, such as species from sect. *Cruciata*, evolve more slowly than shorter annuals, such as species from sect. *Chondrophyllae* [41, 57]. In addition, the effectiveness of DNA barcoding is related to the phylogenetic relation between species; DNA barcoding is more powerful in distantly related taxa and less effective in recently radiated groups [58]. Limited sampling in sect. *Chondrophyllae* in this study (10/163 species belonging to 5 series) may have caused over-estimation of the discriminatory power of DNA barcoding. In contrast, 8/12 species in sect. *Cruciata* were sampled and several closely related species were included, such as *G*. *daurica*, *G*. *officinalis*, *G*. *macrophylla* and *G*. *decumbens* [26].

Natural hybrids and polyploidization pose a great challenge to attempts to barcode species. Hybridization events have been reported in sect. *Cruciata*. Hybridization may have occurred between *G*. *officinalis* and *G*. *daurica* [59, 60], which were used in the present study. All vouchers of these two species shared identical sequences among the chloroplast regions except *matK*, which had two differences (S2 Table); furthermore, two hybrids (*G*. *officinalis*_018 and *G*.*officinalis*_048_1) were found in the ITS region. Additional studies sampling from more populations are needed to confirm this hypothesis.

### The potential application of DNA barcoding in *Gentiana*

Although limited species resolution was achieved in *Gentiana*, DNA barcoding enabled us to confidently assign most individuals at the genus and section level, even using only the *rbcL/matK* region or when the data set was expanded using GenBank sequences. If the section and geographical information of specimens is known, DNA barcoding will make the identification of most *Gentiana* species much easier for non-experts, which will greatly reduce the time and labor compared with morphological identification, especially in species-rich areas [42, 61].

**Fig 3:**
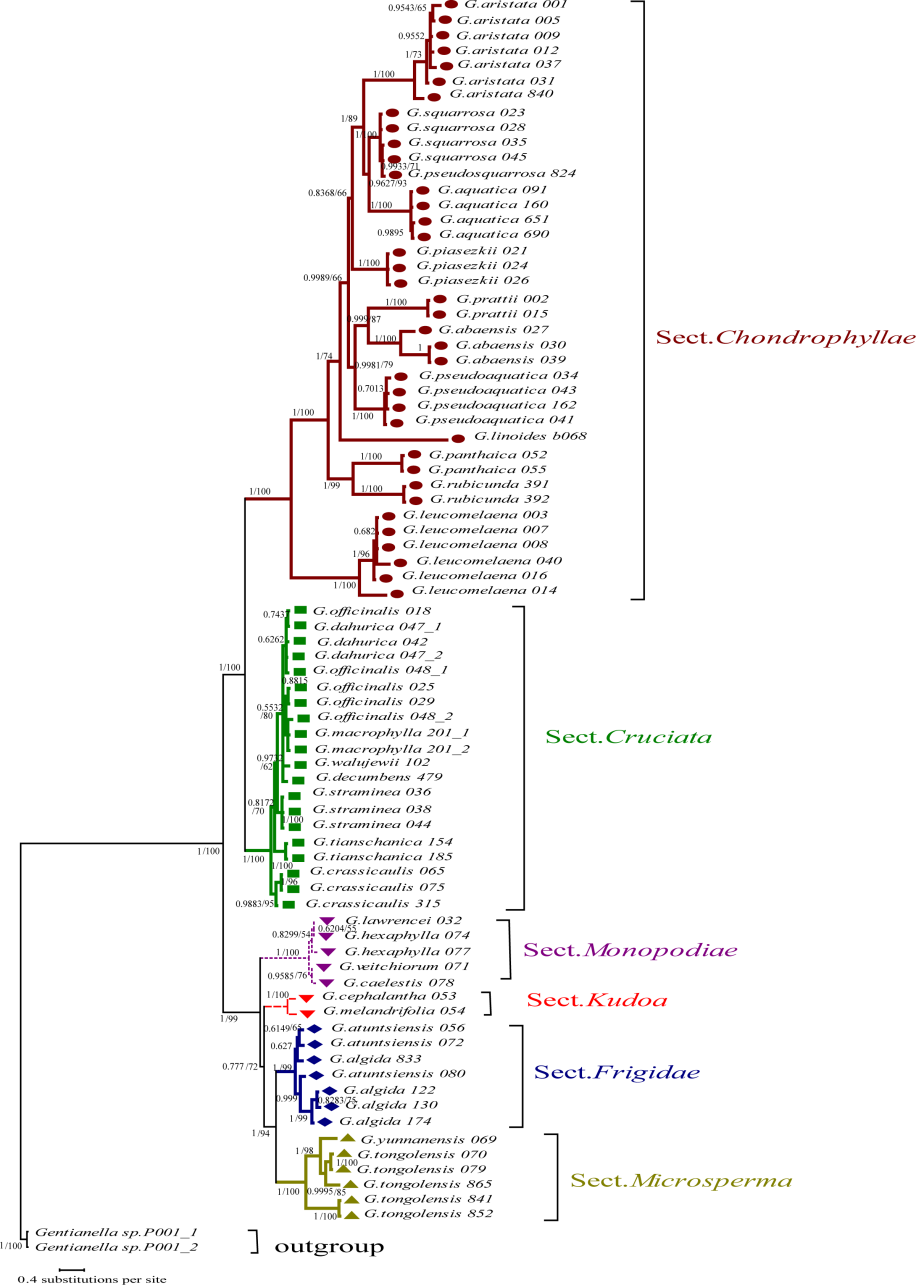
Phylogenetic tree based on Bayesian analysis of *rbcL* + *matK* + ITS + *trnH-psbA*. Bootstrap value ≥ 50% in the NJ analysis and posterior probabilities ≥ 0.95 in the BI analysis are shown on the left and right of the slash, respectively.

Although many have argued that DNA barcoding can be used for species determination [8], studies generally support its use to clarify questions regarding the taxonomy of some groups instead, such as resolving taxonomic uncertainties in *Lysimachia* [9] and *Primula* [17], and revising the taxonomic status of a variety in *Roscoea* [62]. In this study, most species in sect. *Chondrophyllae* were monophyletic, except *G*. *squarrosa* and *G*. *pseudosquarrosa*. All accessions of both species always clustered together with high confidence in the NJ tree and Bayesian analysis (Fig 3). This result may have been caused by many factors, such as imperfect taxonomy, misidentification, introgression or the low discrimination ability of DNA barcoding. According to the description in “A worldwide monograph of *Gentiana*” [23], the morphological characters distinguishing these species are the relative length of the corolla vs. calyx and the color of the seeds. We checked the holotypes of the two species and found that the length of the corolla was largely related to the extent of flowering. Both holotypes have a short corolla in their early blooming stage and a much longer corolla on blooming flowers. Therefore, the morphological data support the conclusion from barcoding that *G*. *pseudosquarrosa* should be treated as a synonym of *G*. *squarrosa*. However, only one specimen of *G*. *pseudosquarrosa* was included in this study, and it is possible that *G*. *pseudosquarrosa* was misidentified due to the small size of calyx and corolla. Additional studies with more samples and more molecular evidence are necessary to validate the taxonomic status of *G*. *squarrosa* and *G*. *pseudosquarrosa*.

DNA barcoding has been widely accepted as a technique to authenticate herbal medicinal materials (e.g. powder, processed roots, barks and leaves) and detect product substitution and contamination [5, 6, 30, 63]. Although authentication of closely related species using DNA barcoding has been challenging [5], DNA barcoding can readily separate species which are morphologically similar but phylogenetically distant. It is very common for morphologically similar products to be used as substitutions in the medicinal plant trade [5]. For example, ‘Qin-Jiao’, the dried roots of four species of *Gentiana* (*G*. *macrophylla*, *G*. *crassicauli*s, *G*. *straminea*, and *G*. *dahurica*) [64, 65], has been a well-known traditional medicinal plant in China for over a thousand years [65]. Adulterants or counterfeits with similar-looking processed roots from other families or genera, such as *Aconitum sinomontanum* (Ranunculaceae, which has the common name “Qin-Jiao” in the Xinjiang Province of China), *Salvia brzewalskii* Maxim (Lamiaceae, called “Hong Qin-Jiao”), and *Veratrilla baillonii* Franch. (Gentianaceae, called “Huang Qin-Jiao”), have entered the commercial market [28, 29]. In order to assess the ability of DNA barcoding to authenticate medicinal herbs, we downloaded *matK* and ITS sequences of these counterfeit species from GenBank and compared them to the correct ‘Qin-Jiao’ sequences from this study using a NJ tree-based method (S4 Fig). The results show that DNA barcoding can successfully differentiate Qin-Jiao from the substitutes. In a previous study [29], ITS2 has been found to be capable of identifying Qin-Jiao adulterants or counterfeits. Our study further verified that DNA barcoding can provide reliable identification and ensure the safety and efficacy of the herbal products from sect. *Cruciata*.

## Conclusions

The suggested core plant barcode (*rbcL + matK*) is not very effective for identifying species in the genus *Gentiana*. Because of poor alignment and frequent inversions, the non-coding region of *trnH-psbA* also may not be desirable as a DNA barcode for *Gentiana*, even though it had the highest genetic variation and relatively high discriminatory performance. ITS was much more effective for species resolution and was capable of discriminating the closely related species in sect. *Cruciata*. A two-region combination of *matK* + ITS is recommended for use as a plant barcode in *Gentiana*. The ITS2 region, with its high sequence recoverability, can serve as a back-up region for ITS. Although DNA barcoding may not always be the perfect identification tool, we emphasize the practical applications of this method in biodiversity surveys, clarifying taxonomic questions and authenticating medicinal plant materials.

## Supporting Information

S1 Fig DNA barcoding identification at the level of genus and section using the GenBank sequence dataset for *Gentiana*. (a-b) NJ trees using *rbcL* and *matK*, respectively, with GenBank sequences for the family of Gentianceae; (c-d) NJ trees using ITS and ITS2, respectively, with GenBank sequences for genus *Gentiana*.
(PDF)

S2 Fig DNA barcoding gap in sect. *Chondrophyllae*. (TIF)

S3 Fig DNA barcoding gap in sect. *Cruciata*. (TIF)

S4 Fig Authentication Qin-Jiao using DNA barcoding. (PDF)

S1 Table Species information and GenBank accession numbers in the genus *Gentiana*. (XLSX)

S2 Table Diagnostic nucleotides in *matK* and ITS regions for *G*. *officinalis* and *G*. *daurica* in sect. *Cruciata*. (DOCX)
