# Supplementary figures and images for: The Use of DNA Barcoding on Recently Diverged Species in the Genus Gentiana (Gentianaceae) in China
Source: PLoS One. 2016 Apr 6;11(4):e0153008. doi: 10.1371/journal.pone.0153008 (PMC4822852; doi:10.1371/journal.pone.0153008)

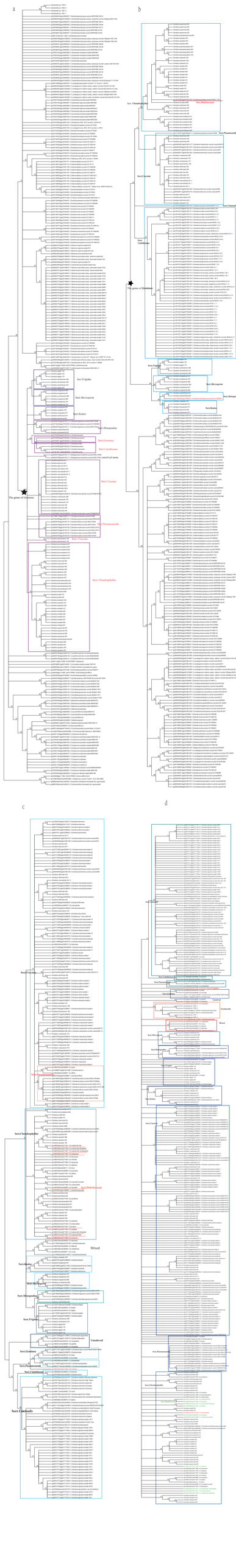

Supplement: S1 Fig — (a-b) NJ trees using rbcL and matK, respectively, with GenBank sequences for the family of Gentianceae; (c-d) NJ trees using ITS and ITS2, respectively, with GenBank sequences for genus Gentiana. (PDF) [file pone.0153008.s001.pdf]

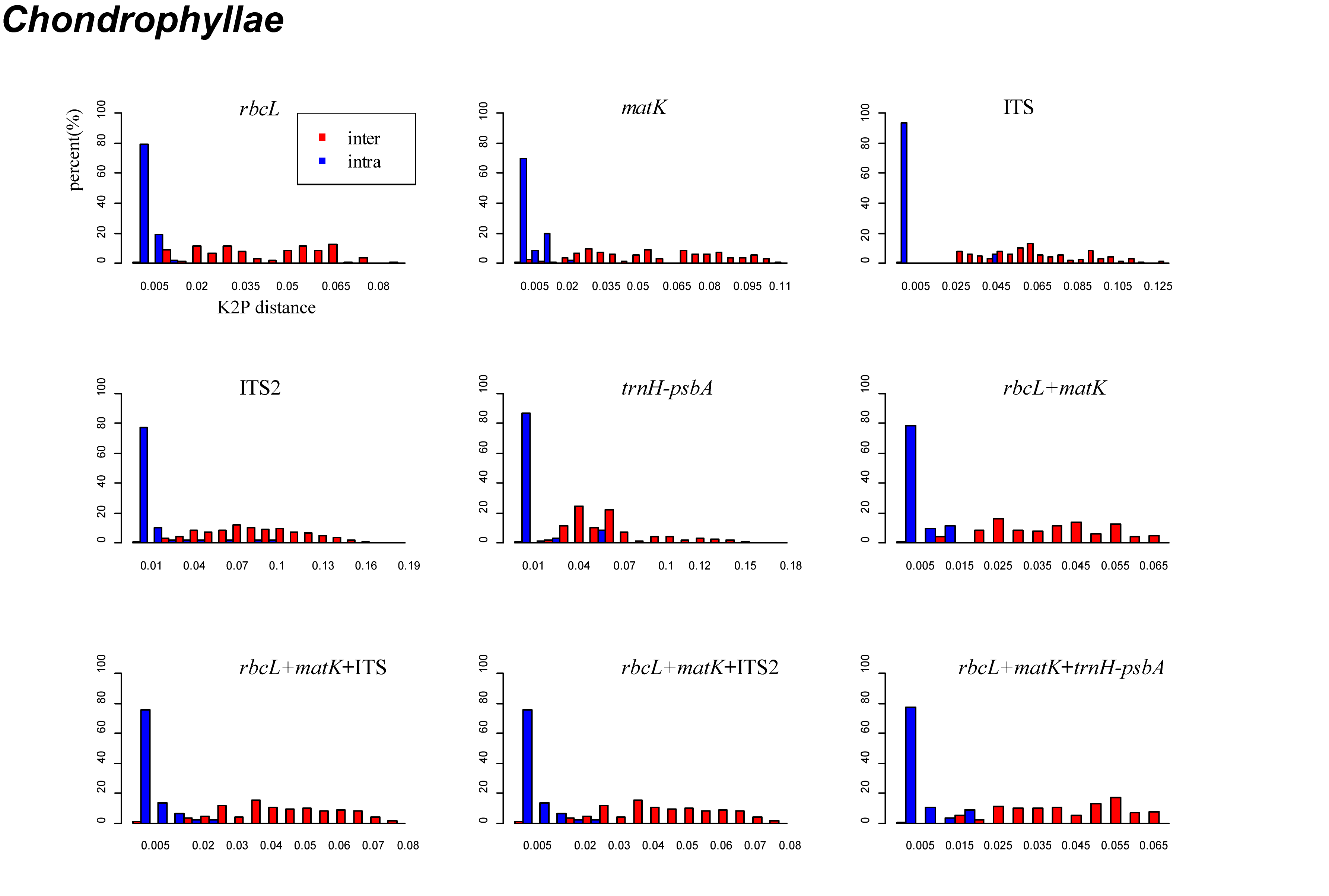

Supplement: S2 Fig — (TIF) [file pone.0153008.s002.tif]

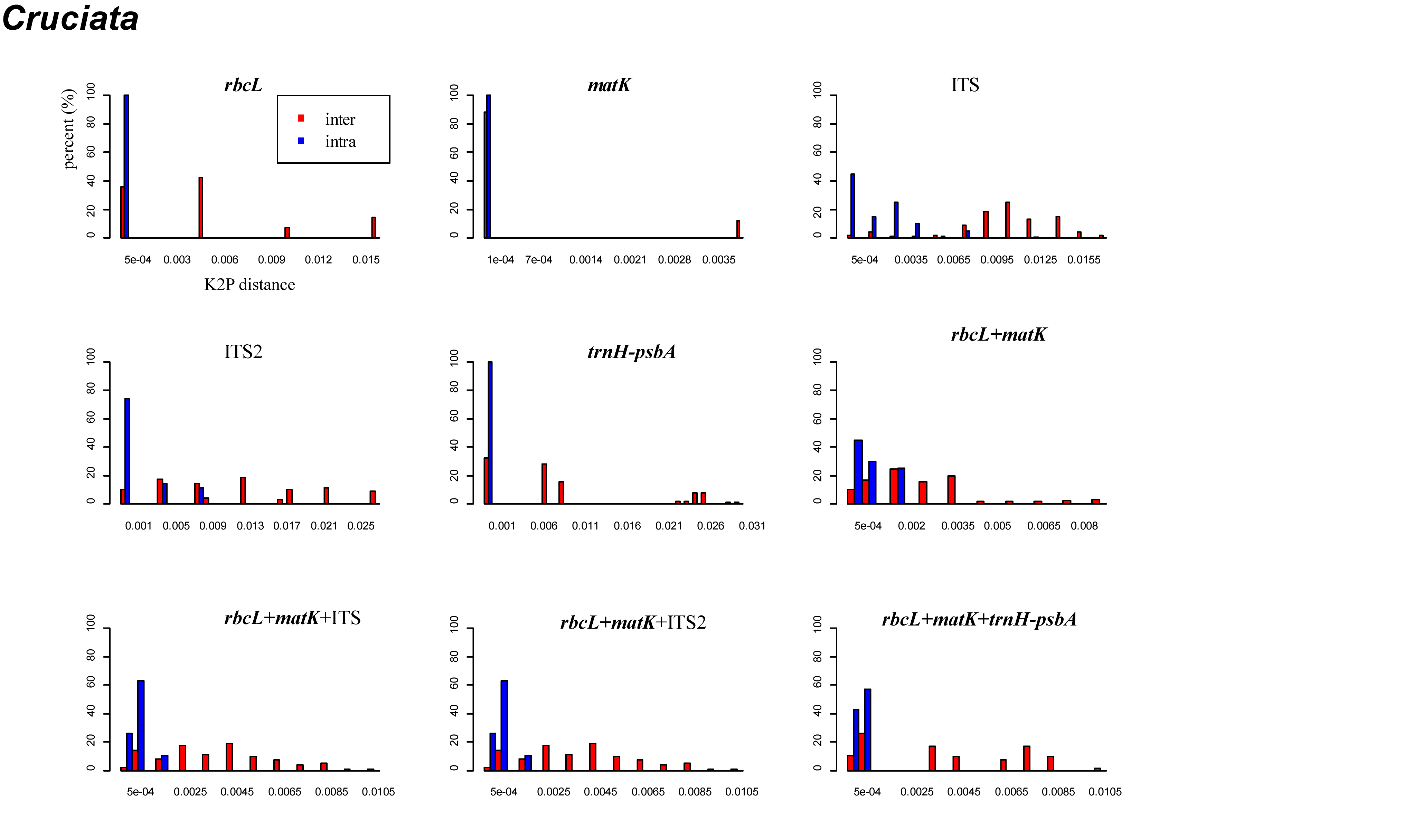

Supplement: S3 Fig — (TIF) [file pone.0153008.s003.tif]

*matK*

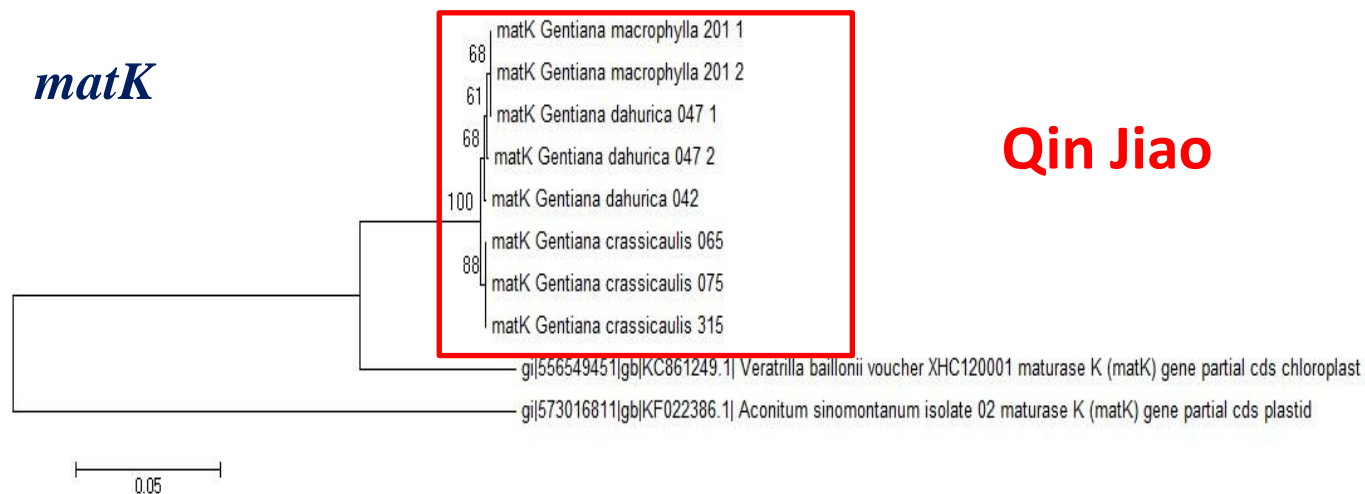

**Qin Jiao**

*ITS*

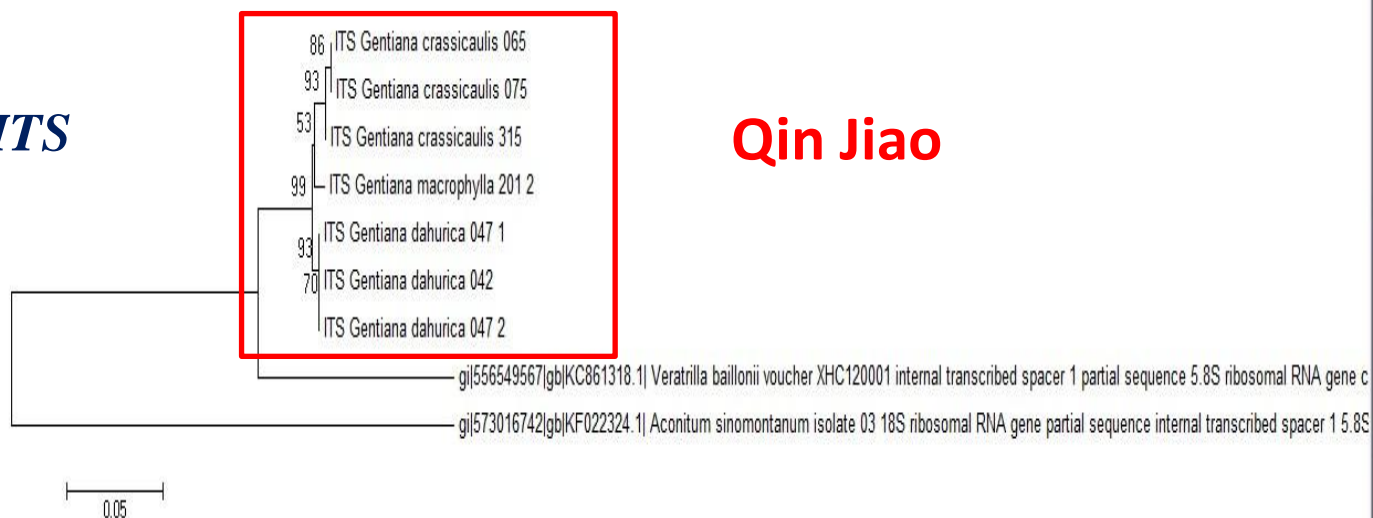

**Qin Jiao**

Supplement: S4 Fig — (PDF) [file pone.0153008.s004.pdf]
