# Supplementary material for: The Use of DNA Barcoding on Recently Diverged Species in the Genus Gentiana (Gentianaceae) in China
Source: PLoS One. 2016 Apr 6;11(4):e0153008. doi: 10.1371/journal.pone.0153008 (PMC4822852; doi:10.1371/journal.pone.0153008)
Supplement: S2 Table — (DOCX) [file pone.0153008.s006.docx]

Table S2 Diagnostic nucleotides in *matK* and ITS regions for *G. officinalis* and *G. daurica* in sect. *Cruciata*

| **Voucher** | ***matK*** | |  | **ITS** | | | | | | | |
| --- | --- | --- | --- | --- | --- | --- | --- | --- | --- | --- | --- |
|  | 95 bp | 144 bp |  | 71 bp | 83 bp | 408bp | 410 bp | 441 bp | 598 bp | 615 bp | 622 bp |
| **G.officinalis_018** | G | T |  | **M** | **R** | **M** | **W** | **Y** | **K** | **S** | C |
| **G.officinalis_025** | T | C |  | A | A | C | T | T | T | G | C |
| **G.officinalis_029** | T | C |  | A | A | C | T | T | T | G | C |
| **G.officinalis_048_1** | T | T |  | **M** | **R** | **M** | **W** | **Y** | **K** | **S** | **Y** |
| **G.officinalis_048_2** | G | T |  | A | A | C | T | T | T | G | T |
| **G.dahurica_042** | T | C |  | C | G | A | A | C | G | C | C |
| **G.dahurica_047_1** | G | T |  | C | G | A | A | C | G | C | C |
| **G.dahurica_047_2** | T | T |  | C | G | A | A | C | G | C | C |
